# The Typhoid Bacillus

**Published:** 1902-10-04

**Authors:** 


### The Hospital.

A Journal of

The Medical Sciences and Hospital Administration.

Vol. XXXIII.—No. 836.] Edited by Sir Henry Burdett, K.C.B., and Solomon C. Smith, M.D., M.R.C.P. [October 4, 1902.

### The Typhoid Bacillus.

We congratulate two distinguished members of the Royal Army Medical Corps, Major Firth and Major Horrocks, upon the accomplishment of a piece of solid scientific work which will do more to raise their own reputations, and to reflect credit upon the distinguished body to which they belong, than any amount of discussion concerning the provisions of Royal Warrants, or concerning the privileges, precedence, or rank of medical as distinguished from combatant officers of the Army. Their paper, which appears in the *British Medical Journal* for last week, is not only a highly important and valuable contribution to both military and general hygiene, but it is also a complete vindication of any claim that may have been made, on the part of the Army Medical Corps, to occupy worthily a conspicuous place in the front rank of the profession of medicine.

We have on several former occasions been called upon to refer to the obscurity which rests upon the channels of transmission of typhoid fever, and upon the high probability, until now unverified by strict experiment, that such transmission might be effected by other agencies than that of water, and especially by flies and dust. The terrible prevalence and fatality of the disease in South Africa has given a stimulus to all inquiries concerning its mode of propagation; and we have more than once called attention to the success of Dr. Leigh Canney in preventing its occurrence in the workers' camps at Assouan, and to his reiterated assertion of his belief that similar means—that is to say, the restriction of the men to recently boiled water—would be equally effective in the conduct of military operations. We are unable to speak from personal knowledge of the conditions which existed at Assouan, and nothing is known of those under which the first case of typhoid usually occurs in an army in tents or on the march. It is obvious that, if the first case could be excluded, there would be no reason to apprehend a second; but the almost universal occurrence of the disease among armies or other great congregations of men is enough to give support to the hypothesis that the bacillus may be widely diffused in a saprophytic form external to the human body, or that it is a modification, brought about by the action of causes common to most camps, of some organism which in its ordinary condition is harmless. Assuming water to be the habitat of the saprophytic form, and that all water is boiled before use, it is of course conceivable that the first case of typhoid may never arise in any given community. Such may have been the case at Assouan, where it is possible that the door may from the first have been effectively closed against the invader. It is equally conceivable, as an abstract proposition, that if soldiers drank nothing but boiled water the first case might not arise in an army in the field. We will not say that the hope of realising such a state of things is utopian, but it does not at present seem to fall within the limits of reasonable probability, and Army surgeons in the future, as in the past, will be likely to find themselves confronted with a condition in which typhoid exists, but in which it cannot be traced with any certainty to its source. The first case would be as impossible to identify as the first star in evening twilight. In such circumstances, it becomes of supreme importance to know in what way the men still in health may be most effectively protected, and it is necessary to remember that all measures of protection involve trouble to the purely military authorities, that they may even interfere with movements which it is desirable to carry out, and that hence, unless they are effectual, they are tolerably certain to be set aside as impracticable by officers in command. If a General were in a position to say to his chief medical officer "See here, the water has been boiled in compliance with your recommendation, and there are fifty fresh cases of typhoid this week," would he not be very likely to add that he would no longer be bothered about a precaution which was manifestly unavailing. Now Majors Firth and Horrocks have shown conclusively that, when typhoid is current in a camp, the free diffusion of its bacillus is accomplished by many other agencies than water. They have shown the bacillus existing for six weeks or more in soils, scattered in dust without any loss of its virulence or its activity, carried by flies from excreta to food, and practically almost omnipresent. Their researches justify the belief that no measures of protection will be effective, when typhoid once exists in a camp or an army, which do not include, in addition to the protection of water, the capture and destruction of the bacillus on its exit from the body—that is to say, more effective methods of dealing with typhoid excreta, including urine, than any which are now in use. They totally condemn the whole principle and practice of "dry earth conservancy," thinking that what it chiefly "conserves" is the bacillus itself; and they recommend a modified water-system for use in camps. With this it might be possible to combine the chemical destruction of the microbe, unless the carriage of the required agents should present an insurmountable difficulty.

